# High proportions of children under 3 years of age consume commercially produced snack foods and sugar‐sweetened beverages in Bandung City, Indonesia

**DOI:** 10.1111/mcn.12764

**Published:** 2019-06-21

**Authors:** Mackenzie Green, Dian N. Hadihardjono, Alissa M. Pries, Doddy Izwardy, Elizabeth Zehner, Sandra L. Huffman

**Affiliations:** ^1^ Helen Keller International New York New York USA; ^2^ Direktorat Gizi Masyarakat‐Kementerian Kesehatan RI Jakarta Indonesia; ^3^ Consultant to Helen Keller International New York New York USA

**Keywords:** child feeding, complementary feeding, double burden, Indonesia, snack food, sugar‐sweetened beverage

## Abstract

Child undernutrition continues to be a national concern in Indonesia, whereas childhood overweight/obesity rises. Economic development has led to wide availability of highly processed foods and beverages, with growing evidence that children are consuming commercial snack products during the critical complementary feeding period. This study assessed the prevalence and patterns of consumption of commercially produced snack foods and sugar‐sweetened beverages among Indonesian children. A cross‐sectional survey was conducted with 495 mothers of children aged 6–35 months living in Bandung City, Indonesia. Among all children, 81.6% consumed a commercial snack food and 40.0% consumed a sugar‐sweetened beverage in the day preceding the interview. At 6–11 months, 46.5% of children consumed a snack food and 2.0% consumed a sugar‐sweetened beverage. Snack foods were consumed 3 or more times a day by 60.0% of children 24–35 months of age. Sweet biscuits and savory snacks were the most commonly consumed snack foods; sweetened milks and sweetened teas were the most common beverages. Maternal education, child age, and consumption of a commercially produced complementary food were associated with snack food consumption. Factors associated with sugar‐sweetened beverage consumption were child age and consumption of a commercially produced complementary food or breastmilk substitute. These findings reflect a high presence of processed, high‐sugar/salt commercial snack products in the diets of children 6–35 months. National attention should focus on interventions to reduce reliance on processed snack products and increase consumption of nutrient‐rich, locally available foods during the complementary feeding period.

**Key messages**

- This study found high and frequent consumption of commercially produced snack foods among children 6–35 months in Bandung City.
- The level of consumption found in this study, coupled with the stagnant levels of child stunting, growing rates of overweight/obesity among the young, and increasing burden of non-communicable disease in Indonesia, is worrisome.
- National attention is needed to address suboptimal complementary feeding practices through improved feeding guidelines, caregiver education, and regulation of promotions and packaging of commercial snack products.

## 1 INTRODUCTION

Suboptimal infant and young child feeding practices persist in Indonesia, and child undernutrition continues to be a national concern (Beal, Tumilowicz, Sutrisna, Izwardy, & Neufeld, 2018; Ministry of Health [MOH], 2015; Ng, Dibley, & Agho, 2012). One‐third (36.6%) of children 6–23 months meet World Health Organization (WHO) recommendations for a minimum acceptable diet 1Defined as children 6–23 months having minimally acceptable feeding frequency, diet diversity, and consumption of breastmilk or other milks (WHO, 2010) for infants and young children (Badan Pusat Statistik [BPS], National Population and Family Planning Board [BKKBN], Kementerian Kesehatan [Kemenkes], & ICF International, 2013; WHO, 2010), and the country has one of the highest rates of child stunting in the world (Black et al., 2008; White et al., 2016). In 2013, 37.2% of under‐five children were stunted, 19.6% were underweight, and 12.1% were wasted, with little improvement from previous years (MOH, 2014). Concurrently, the prevalence of overweight and obesity has been rising among young Indonesian children (MOH, 2013; Rachmi, Agho, Li, & Baur, 2016; Rachmi, Li, & Baur, 2017). Government estimates from 2013 reported a combined total of 11.9% overweight/obese in children under five (MOH, 2013), with increases particularly evident in urban areas and in Java (Rachmi et al., 2016; Rachmi et al., 2017).

Indonesia, like many low‐ and middle‐income countries (LMIC), is undergoing a nutrition transition (Shrimpton & Rokx, 2013; Vaezghasemi, 2017), with the traditional diet shifting towards a westernized diet as the country experiences economic and social development (Popkin, Adair, & Ng, 2012). These westernized dietary patterns are marked by higher intakes of animal‐source foods, added sugars and fat, and highly processed foods. Moreover, increased global trade of foods has expanded the availability of processed foods in LMIC, which are distributed through widening networks of supermarkets and minimart chains, predominantly in urban areas (Baker & Friel, 2016; Popkin et al., 2012; Shrimpton & Rokx, 2013). A 2015 Indonesian consumer insights report found increased household spending on beverage and packaged foods across all income levels, with spending on packaged foods ranging from 18 to 32% of monthly household expenditure (Deloitte, 2015).

Indonesia is also one of many LMIC where snack food consumption among children is prevalent. A study of rural children 1–12 years found that 68 (30.7%) of the 221 different foods they consumed were snacks (Sekiyama, Roosita, & Ohtsuka, 2012) and West Java research among 2‐ to 5‐year‐old children noted the popularity of traditional snacks (Nirmala, Februhartanty, & Wiradnyani, 2016). White et al. (2016) studied consumption in children 6–24 months in East Java and found 54.0% ate commercial biscuits, fried snacks, cakes, or sweets in the last 24 hours. Consumption of snack products—often high in sugar, salt, trans fats, and nutrient‐poor (Gupta, Downs, Ghosh‐Jerath, Lock, & Singh, 2016; Monteiro, Levy, Claro, de Castro, & Cannon, 2011; Moodie et al., 2013)—during the complementary feeding period is concerning as they can displace breastmilk and nutritious foods in the diet, leading to deficiencies in micronutrients and macronutrients (Anderson, Cornwall, Jack, & Gibson, 2008; Kimmons et al., 2005; Tzioumis, Kay, Wright, & Adair, 2015; Vartanian, Schwartz, & Brownell, 2007) during a critical window for optimum growth and development (Shrimpton et al., 2001).

Although research has documented snack consumption in Indonesia, primarily among older children and with traditional snacks, there is limited evidence on consumption of the widely available commercially produced snack products among infants and young children. Given the suboptimal infant and young child feeding practices, burgeoning overweight/obesity rates among young children, and the changing food system in Indonesia making commercial foods easily accessible, there is a critical need to understand consumption during the complementary feeding period. The objective of this research was to assess the prevalence and patterns of consumption of commercially produced snack foods and sugar‐sweetened beverages (SSB) among children under 3 years of age in Bandung City, Indonesia.

## 2 METHODS

### 2.1 Study design and study population

This was a cross‐sectional study with mothers of children aged 0–35 months in Bandung City, Indonesia. Building on a series of studies which documented high consumption of commercial food products among young children in the largest metropolitan area of Cambodia, Nepal, Senegal, and Tanzania (Pries et al., 2017), our interest was to assess consumption in a large urban area outside of Jakarta, the national capital. Bandung City is the fourth largest city in Indonesia and capital of West Java Province (BPS Kota Bandung, 2014), a province with a high level of under‐five stunting (35.3%; MOH, 2013). We included children up to 35 months as the Codex Guidelines on Formulated Complementary Foods for Older Infants and Young Children defines young children as those up to 3 years of age (Food and Agriculture Organization of the United Nations & WHO, 2013).

A two‐stage cluster sampling procedure was used to obtain a representative sample of mothers living and seeking child health services in Bandung City. Health facilities served as a proxy to reach the general population as child health service utilization is high in urban West Java: 91.2% and 85.6% of children 12–23 months of age received DPT3 and measles immunizations (BPS et al., 2013). Mothers of children 0–35 months residing and seeking child health services in Bandung City were eligible if they met study criteria: (a) her child was not severely ill, (b) she was the biological mother of the child, (c) the child was from a singleton birth, and (d) at delivery the child was not in the neonatal intensive care unit (NICU) and the mother did not experience severe delivery complications. The rationale for these criteria was that the study gathered information on antenatal care, delivery, and breastfeeding, which only biological mothers could answer, and twins/multiple births, delivery complications, and NICU can impede or delay breastfeeding behaviours.

### 2.2 Sample size

The sample size for this study was 594 children 0–35 months of age, calculated to detect a 60.0% prevalence of children 6–23 months consuming commercial snack foods on the preceding day with a 0.95 level of confidence, a margin of error of 0.80, and a design effect of 2 to account for cluster sampling. Studies in Cambodia and Nepal documented prevalence of 55.0% and 74.1% among similarly aged children (Pries, Huffman, Mengkheang, et al., 2016; Pries, Huffman, Adhikary et al., 2016) and White et al. (2016) found that 54.0% of children 6–24 months of age in East Java consumed an *unhealthy* snack. Results presented here are for 495 children 6–35 months of age because few children under 6 months received complementary foods.

### 2.3 Sampling procedure and data collection

A list of public and private facilities providing child health services in Bandung City was obtained from the Bandung City Health Office, along with the number of under‐five child health service visits (immunization and outpatient) in the public sector facilities for 2016. Private facilities were contacted by the study team to obtain similar utilization statistics for 2016. Community‐based services, such as *posyandu*, were excluded as their schedules, locations, and participation levels are highly variable. The average number of child health visits per month per facility was calculated, and a sampling frame was generated with facilities having 100 or more child health visits per month. This cut‐off was chosen for feasibility of logistics. A total of 33 clusters were allocated across the sampling frame through probability proportional to size, with the average monthly visits as the measure of size. The number of clusters was informed by NetCode 2Network for Global Monitoring and Support for Implementation of the International Code of Marketing of Breast‐milk Substitutes and Subsequent Relevant World Health Assembly Resolutions methodology (WHO & United Nations International Children's Emergency Fund [UNICEF], 2017). Fourteen public facilities and 10 private facilities were selected, with multiple clusters allocated to larger facilities. Within each cluster, 18 mothers were interviewed, recruited equally across 6‐month age groups (0–5.9 months, 6.0–11.9 months, 12.0–17.9 months, 18.0–23.9 months, 24.0–29.9 months, and 30.0–35.9 months) with three mothers per age group, per cluster.

A field team was trained on study procedures, questionnaire content, and research ethics. Training involved one week of classroom learning, followed by a second week of practice in two health facilities unrelated to the study. Interviews took place between January and March 2018. Facilities were notified approximately one week before data collection began. At a facility, study recruiters approached every woman waiting for child health services. Mothers with children under three years of age, either accompanying her or at home, were then screened for Bandung City residence and eligibility to participate. If no additional interviews were needed for the child's age group at that facility, the mother would not be recruited. Eligible mothers were then assigned to enumerators for interview in Bahasa Indonesia. If mothers were away from their child more than four hours in the preceding day, enumerators attempted to call other caregivers during the interview to complete the questions on food/beverage consumption.

This study was reviewed and approved by the Ethics Committee, Faculty of Medicine, Universitas Padjadjaran, Bandung City, Indonesia, and written informed consent was obtained from all participants.

### 2.4 Questionnaire design

A structured questionnaire used previously in Cambodia, Nepal, Senegal, and Tanzania (Feeley et al., 2016; Pries, Huffman, Mengkheang, et al., 2016; Pries, Huffman, Adhikary et al., 2016; Vitta et al., 2016) was adapted for the Indonesian context and pretested for clarity, relevance, completeness, and flow of interview with mothers in two health facilities unrelated to the study. Background characteristics collected for mothers included: age, parity, marital status, education, work status, and separation from her child in the preceding day. Mothers were asked if they had read, heard, or seen an advertisement, sign/banner, display, price discount, or other commercial promotion for snack products since the birth of their child. Information was recorded on the child's date of birth, sex, and if the child was sick or had different dietary intake in the preceding day. Data were captured on the number of household members, and a simplified subset of household asset questions from the most recent Indonesia Demographic Health Survey (IDHS; BPS et al., 2013) were asked to measure relative household wealth. These 10 questions were identified by EquityTool through principal component analysis to provide a simplified index for comparing the wealth profile of a sample to the national urban population in the IDHS 2012 (Kappa 0.755 with national urban population; EquityTool, 2018), and the simplified approach has been shown to be both highly reliable and valid (Chakraborty, Fry, Behl, & Longfield, 2016; Ergo, Ritter, Gwatkin, & Binkin, 2016).

Data to assess breastfeeding and complementary feeding practices in the last 24 hours were collected according to WHO methods in Indicators for Assessing Infant and Young Child Feeding Practices (WHO, 2010), with an adapted approach to record each food and time of day it was consumed during the dietary free recall by the mother. Standardized questions were also asked to determine the consumption of commercially produced snack foods and SSB in (a) the day prior to interview and (b) the week prior to interview. If a child consumed any of these products in the prior week, mothers were asked to report on the frequency of weekly consumption using methods by Faber and Benadé (2007) and to spontaneously provide the main reason the child ate/drank these commercial products.

Commercially produced snack foods and SSB were considered any manufactured and packaged products intended for general consumption, not specifically formulated for young children (Pries, Huffman, Mengkheang, et al., 2016), and categories of interest were identified from previous literature (Pries et al., 2017) and in consultation with local experts. Snack food categories included the following: (a) sweet biscuits, cookies, and wafers; (b) savory (salty) snacks, chips/crisps, puffs, and *krupuk* (shrimp chips); (c) sweet cake, brownies, and doughnuts; (d) candy, chocolate, and jellies/*agar‐agar*; (e) ice cream; and (f) instant noodles. SSB included (a) packaged juice/juice drinks, (b) soda or carbonated/soft drinks, (c) sweetened milks, and (d) commercial sweetened tea. Sweetened milks were milks marketed for general consumption, or for children older than three years, with added sugar and flavours, excluding sweetened condensed milk. In addition to snack products, consumption of breastmilk substitutes (BMS) and commercially produced complementary foods (CPCF) was also measured. BMS was defined as any formula, milk, or milk‐like product marketed as suitable for feeding children younger than three years of age (WHO & UNICEF, 2017). CPCF were classified as any foods recommended for introduction at an age less than three years or labelled with words like *baby*, *toddler*, or a synonym (WHO & UNICEF, 2017).

The questionnaire was translated into Bahasa Indonesia and then back‐translated into English to check for accuracy. Data were collected electronically with Samsung mobile tablets and the Open Data Kit (ODK) application. Completed questionnaires were uploaded to an online data platform (ONA, 2018) each night, and twice‐weekly data quality checks were run.

### 2.5 Statistical analyses

All data were cleaned and analyzed using Stata version 14 (StataCorp, College Park, TX, USA). A continuous wealth index score relative to the study sample was generated using procedures developed by EquityTool (EquityTool, 2018). Using this score, households were allocated to the urban wealth quintiles reported in the 2012 IDHS for comparison to a national sample. For regression analysis, households were categorized into terciles based on this score, for within‐in sample comparisons. The mother's time away from her child was categorized for analysis using the median value of hours away. Current breastfeeding was defined as the child receiving breastmilk in the previous day. Continued breastfeeding at one and two years were calculated according to WHO Indicators for Assessing Infant and Young Child Feeding Practices (WHO, 2010). Dichotomous variables for consumption in the preceding day were generated and coded *yes* if a mother reported consumption in the previous day or during the free diet recall. Frequency of consumption for snack foods in the preceding day was measured by the number of individual times a mother reported her child eating the food during the free recall and was categorized for analysis. SSB data were not captured in the free recall.

Descriptive analyses calculated percentages for categorical variables, and means and standard deviations for continuous variables. Differences between age groups were assessed using two‐sided Pearson's chi‐square tests. Logistic regression controlling for clustering at the health facility was used to assess the association of maternal, child, and household characteristics with consumption of any snack food and any SSB in the preceding day. The unadjusted association of each characteristic with each consumption outcome was measured with bivariate logistic regression. The unadjusted odds ratio, 95% confidence interval, and *p*‐value are reported.

A full multivariable adjusted model was developed for each consumption outcome and covariates having an unadjusted bivariate association of *p* ≤ 0.05. Categorical variables were assessed for inclusion using their overall *p*‐value for the variable. Covariates were checked for multicollinearity using variance inflation factor; no interaction between covariates was assessed for simplicity. Adjusted odds ratios and 95% confidence intervals are reported for the full models. Goodness of fit was determined through the Hosmer‐Lemeshow test with *p* = 0.8768 for snack food consumption and *p* = 0.5763 for SSB consumption.

## 3 RESULTS

### 3.1 Study population

**Figure 1:**
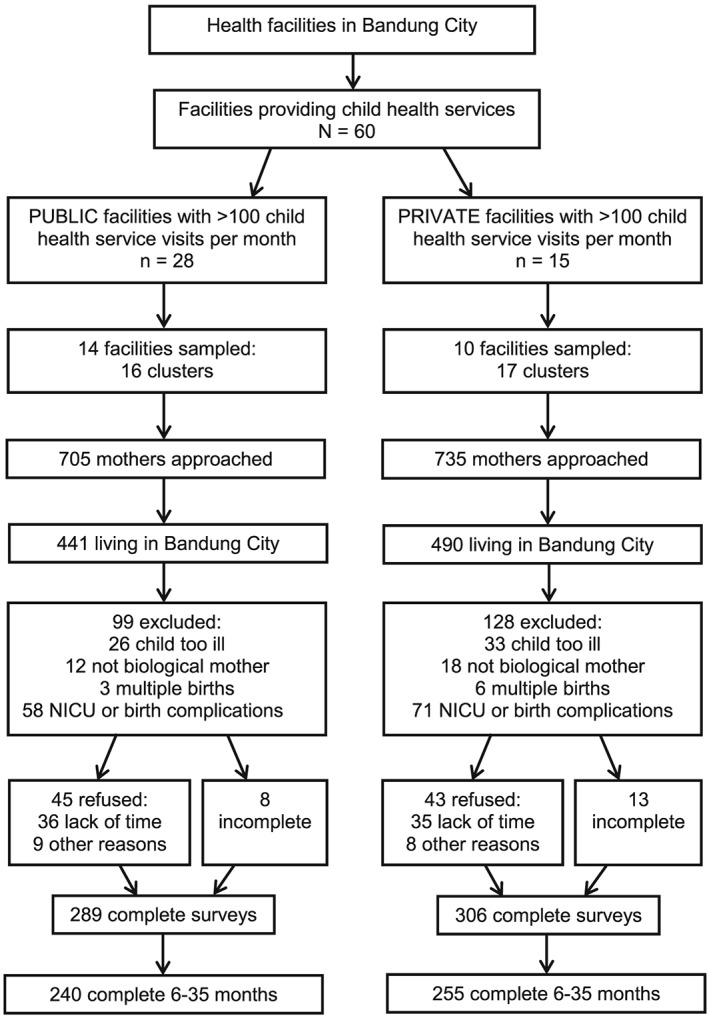
Sampling profile for facilities and mothers of children 0–35 months of age. Out of 27 private facilities, *n* = 7 refused to provide statistics on health service visits per month

Among mothers of children 0–35 months of age, 595 surveys were completed. See Figure 1 for the sampling profile. This analysis includes only the 495 children 6–35 months of age, 255 (51.5%) from the private sector, and 240 (48.5%) from the public sector.

### 3.2 Background characteristics

Background characteristics for mothers, children, and their households are summarized in Table 1 for the subsample of children 6–35 months of age. Mothers were on average 30 years old and all had attended school, with 79.2% completing secondary level or higher. The majority of households were wealthy as compared to the 2012 IDHS, with 76.8% in the two wealthiest national urban quintiles (BPS et al., 2013).

**Table 1:** Mother, child, and household background characteristics^a^

Characteristics	Mothers with children 6–35 months (*n* = 495)
**Mother**	
Age (years)	29.9 ± 5.5
Parity	
1	37.8 (187)
2	36.8 (182)
≥3	25.5 (126)
Marital status (Married)	97.8 (484)
Highest level of education	
Elementary school	4.4 (22)
Junior high school	16.4 (81)
Senior high school	43.0 (213)
Diploma	13.3 (66)
University	22.8 (113)
Any work in the past month	30.9 (153)
Hours away from child preceding day	
0	83.2 (412)
>0 to <8	6.1 (30)
≥8	10.7 (53)
Reported exposure to commercial snack food promotion	97.6 (483)
Reported exposure to SSB promotion	95.8 (474)
**Child**	
Age (months)	
6.0–11.9	20.0 (99)
12.0–17.9	20.0 (99)
18.0–23.9	20.0 (99)
24.0–29.9	20.0 (99)
30.0–35.9	20.0 (99)
Sex (male)	54.1 (268)
Sick preceding day	63.2 (313)
**Household**	
Number of members	5.0 ± 1.8
National urban wealth quintiles, IDHS 2012^b^	
1	1.4 (7)
2	3.8 (19)
3	18.0 (89)
4	36.0 (178)
5	40.8 (202)
Wealth terciles	
Lowest wealth	32.3 (160)
Middle wealth	31.9 (158)
Highest wealth	35.8 (177)

*Note*. SSB, sugar‐sweetened beverage.

a Data presented as percentage (*n*) or mean ± standard deviation.

b Indonesia Demographic Health Survey (IDHS). Quintile 1, lowest wealth; Quintile 5, highest wealth (BPS et al., 2013).

Because mothers were recruited at child health services, they were asked if in the preceding day their child was sick and how their dietary intake may have differed. Nearly two‐thirds of children (63.2%) were reported sick. About half of all children (48.7%) ate *less food* than the prior day, whereas 36.6% had the *same amount*, and 14.8% ate *more food*; a quarter of all children (24.2%) had different foods than normal.

### 3.3 Breastfeeding, complementary feeding, and consumption of commercial snack products

Breastfeeding was nearly universal, and current breastfeeding remained high through 23 months of age (Table 2); the percentage of women breastfeeding dropped significantly after 2 years (*p* < 0.001, 18–23 months compared with 24–29 months). Consumption of commercial infant and young child feeding products was high in the day prior to the survey: 49.5% of children received a BMS and 37.4% a CPCF.

**Table 2:** Breastfeeding and consumption of commercial snack food and beverage products in the preceding day and week^a^

Breastfeeding status	
Ever breastfed^b^	99.2 (491)
Current breastfeeding (months)	
6.0–11.9	84.9 (84)*
12.0–17.9	73.7 (73)
18.0–23.9	76.8 (76)***
24.0–29.9	29.3 (29)**
30.0–35.9	10.1 (10)
Continued breastfeeding at 1 year^c^	72.7 (40)
Continued breastfeeding at 2 years^d^	71.9 (41)
Continued breastfeeding at 3 years^e^	11.3 (7)

Consumption of commercial snack food and beverage products on preceding day and week by child age						
		6.0–11.9 mo (*n* = 99)	12.0–17.9 mo (*n* = 99)	18.0–23.9 mo (*n* = 99)	24.0–29.9 mo (*n* = 99)	30.0–35.9 mo (*n* = 99)
Commercial snack food						
Preceding day		46.5 (46)***	84.8 (84)	89.9 (89)	94.9 (94)	91.9 (91)
Preceding week		61.6 (61)***	91.9 (91)*	98.0 (97)	100 (99)	100 (99)
Commercial sugar‐sweetened beverage						
Preceding day		2.0 (2)***	22.2 (22)***	49.5 (49)*	65.7 (65)	60.6 (60)
Preceding week		4.0 (4)***	37.4 (37)***	73.7 (73)*	86.9 (86)	79.8 (79)

a Data presented as percentage (*n*).

b Among children 6.0–35.9 months (*n* = 495).

c Among children 12.0–15.9 months (*n* = 55).

d Among children 20.0–23.9 months (*n* = 57).

e Among children 32.0–35.9 months (*n* = 62).

* Significant difference between age group and next oldest age group at *p* < 0.05.

** Significant difference between age group and next oldest age group at *p* < 0.01.

*** Significant difference between age group and next oldest age group at *p* < 0.001.

The majority of children 6–35 months (81.6%) consumed any commercial snack food in the preceding day and 40.0% any SSB. Consumption on the preceding day increased with age, with nearly half (46.5%) of children 6–11 months consuming a snack food compared with almost all (91.9%) children 30–35 months (*p* < 0.001). Consumption of any SSB also increased significantly with age over 6 to 29 months (*p* < 0.001, 6–11 compared with 24–29 months), although overall prevalence of consumption was less than with snack foods. Reported consumption of commercial snack products in the week prior to interview was higher than in the preceding day for both snack foods and SSB, and weekly consumption also increased with age.

**Figure 2:**
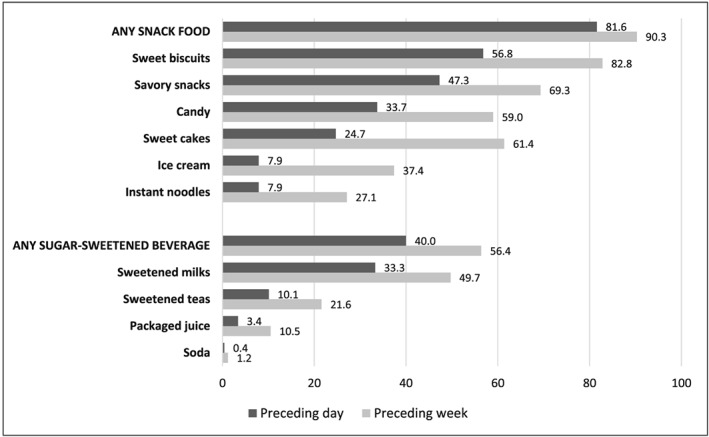
Percentage of children 6–35 months consuming commercial snack foods and sugar‐sweetened beverages in the preceding day and week

Figure 2 shows the percentage of children 6–35 months who consumed the different types of commercial snack foods and SSB in the day and week prior to the interview. Sweet biscuits and savory snacks were the most commonly consumed foods in both timeframes. Sweetened milk was the most commonly consumed SSB; soda or carbonated/soft drinks were rarely consumed among children in this sample.

### 3.4 Frequency of consumption of commercial snack products

**Figure 3:**
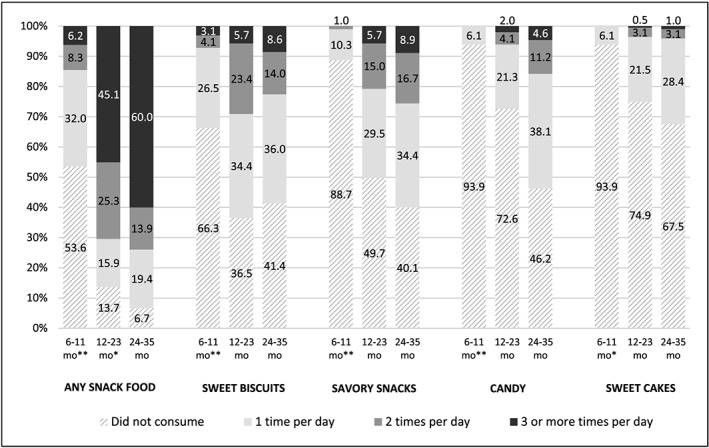
Percentage of children 6–35 months consuming commercial snack foods 1 time, 2 times, or 3 or more times in the previous day, by age of child. Significant difference between age group and next oldest age group at: *p < 0.01, **p < 0.001. Number of children per age group: 6.0–11.9 months *n* = 99; 12.0–23.9 months *n* = 198; 24.0–35.9 months *n* = 198. Frequency data not available for mothers who reported consumption in survey but not in diet recall and *n* = 2 children who did not receive any foods in the preceding day; missing *n* = 36 any snack food, *n* = 19 sweet biscuits, *n* = 13 savory snacks, n = 3 candy, and *n* = 5 sweet cakes. Due to rounding, percentages may not add up to 100%

Figure 3 shows the frequency of consumption of any commercial snack food and the four types most commonly consumed, by age group in the preceding day. No children 6–11 months received ice cream in the preceding day, 5.6% consumed it one time at 12–23 months, and 13.3% one time at 24–35 months (*p* < 0.02 difference between each age group). Instant noodle consumption was similarly infrequent but increased significantly between 12–23 months and 24–35 months (*p* = 0.004): at 6–11 months, 99.0% did not consume, and 1.0% consumed one time; at 12–23 months, 95.4% did not consume, and 4.6% consumed one time; and at 24–35 months, 85.8% did not consume, 13.7% consumed one time, and 0.5% consumed two times.

**Figure 4:**
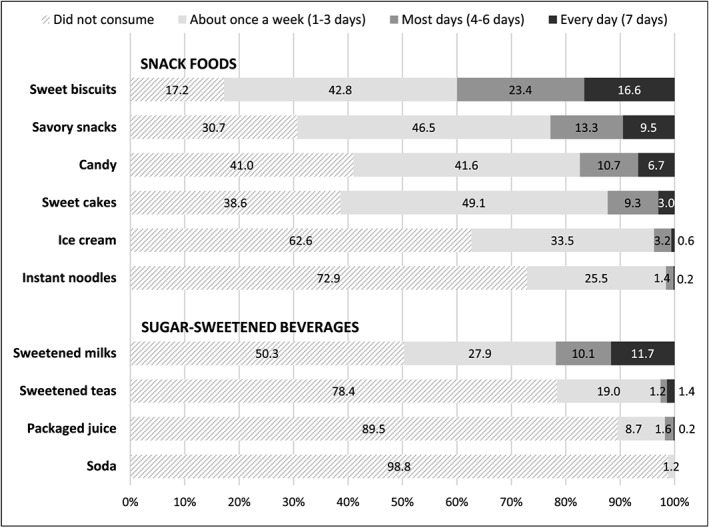
Frequency of consumption of commercial snack foods and beverages in the past week among children 6–35 months of age. Due to rounding, percentages may not add up to 100%

For children who consumed snack foods and SSB in the past week, mothers were asked how frequently her child consumed the product and Figure 4 shows the responses mothers provided. Sweet biscuits, savory snacks, and sweetened milks were consumed most frequently over the week, with nearly 10% or more of all children consuming them every day.

### 3.5 Reason for use of commercial snack products

Mothers were asked to provide the one main reason why her child received a commercial snack food or a SSB in the previous week. Of mothers with children consuming products in the past week, 38.0% said the child received the snack food and 48.4% said the child received the SSB, because her child asks/wants/demands it. Another 33.6% and 32.3% said the snack food or SSB make the child happy. Other main reasons were reported by less than 10% of mothers whose children consumed products in the last week and differed little between snack foods and SSB. Rarely did mothers consider the snack products *healthy*/*good for the child's development* (2.5% snack food, 6.1% SSB).

### 3.6 Characteristics associated with use of commercial snack foods and sugar‐sweetened beverages

Results from bivariate and multivariable logistic regression to assess the characteristics associated with consumption in the preceding day are shown in Tables 3 and 4. In the adjusted multivariable model, three factors were found significantly associated with consumption of a commercial snack in the preceding day. Children of less educated mothers were 2.03–3.93 times more likely to have consumed a snack food compared with mothers with post‐secondary level of education. Older children were more likely to have eaten a snack food, as compared with younger children. Consumption of a CPCF in the day prior to the survey was negatively associated with snack consumption. In the adjusted multivariable model for SSB, the likelihood of consuming a SSB was also reduced if the child received a CPCF, as well as a BMS, in the prior day. Older children were more likely to drink a SSB in the prior day compared with younger children.

**Table 3:** Bivariate and multivariable logistic regression of commercial snack food consumption in previous day among children 6–35 months^a^ (*n* = 495)

Variable	Snack consumed	Unadjusted bivariate^b^			Adjusted multivariable^c^		
	% (*n*)	OR	(95% CI)	*p*‐value^d^	OR	(95% CI)	*p*‐value^d^
**Maternal factors**							
Maternal age (year)		1.01	(0.98, 1.05)	0.477	‐	‐	‐
Parity							
Primaparous	77.5 (145)	1			‐	‐	‐
Multiparous	84.1 (259)	1.53	(0.93, 2.52)	0.093	‐	‐	‐
Maternal education							
Elementary, Junior high	90.3 (93)	3.12	(1.35, 7.21)	0.002	3.93	(1.39, 11.10)	0.009
Senior high	83.1 (177)	1.65	(1.13, 2.41)		2.03	(1.30, 3.18)	
Diploma, University	74.9 (134)	1			1		
Any work in past month							
Yes	77.1 (118)	0.66	(0.43, 1.00)	0.054	0.62	(0.38, 1.03)	0.067
No	83.6 (286)	1			1		
Hours away from child preceding day							
0	82.5 (340)	1			‐	‐	‐
>0 to <8	83.3 (25)	1.06	(0.42, 2.68)	0.237	‐	‐	‐
≥8	73.6 (39)	0.59	(0.32, 1.09)		‐	‐	‐
Reported exposure to snack food promotion							
Yes	81.6 (394)	0.86	(0.23, 3.46)	0.861	‐	‐	‐
No	83.3 (10)	1			‐	‐	‐
**Child factors**							
Child age (months)							
6.0–11.9	46.5 (46)	1			1		
12.0–17.9	84.9 (84)	6.45	(3.60, 11.55)	<0.001	5.27	(2.17, 12.82)	<0.001
18.0–23.9	89.9 (89)	10.25	(4.69, 22.41)		6.99	(2.61, 18.76)	
24.0–29.9	95.0 (94)	21.66	(9.78, 47.96)		10.56	(3.56, 31.32)	
30.0–35.9	91.9 (91)	13.11	(5.73, 29.98)		6.17	(1.74, 21.93)	
Child sex							
Male	81.0 (217)	0.91	(0.57, 1.46)	0.696	‐	‐	‐
Female	82.4 (187)	1			‐	‐	‐
Sick preceding day							
Yes	79.9 (250)	0.72	(0.45, 1.16)	0.179	‐	‐	‐
No	84.6 (154)	1			‐	‐	‐
Amount ate preceding day versus normal							
Same amount	82.3 (149)	1			‐	‐	‐
Less food	80.5 (194)	0.89	(0.54, 1.46)	0.838	‐	‐	‐
More food	82.6 (61)	1.09	(0.65, 1.84)		‐	‐	‐
Ate different foods preceding day versus normal							
Yes	84.2 (101)	1.26	(0.73, 2.18)	0.402	‐	‐	‐
No	80.8 (303)	1			‐	‐	‐
Breastfed preceding day							
Yes	76.1 (207)	1			1		
No	88.3 (197)	2.38	(1.43, 3.96)	0.001	0.90	(0.42, 1.96)	0.800
BMS preceding day							
Yes	81.2 (199)	0.95	(0.55, 1.65)	0.854	‐	‐	‐
No	82.0 (205)	1			‐	‐	‐
CPCF preceding day							
Yes	60.5 (112)	0.90	(0.05, 0.16)	<0.001	0.15	(0.08, 0.29)	<0.001
No	94.2 (292)	1			1		
**Household factors**							
Number household members		1.00	(0.90, 1.12)	0.998	‐	‐	‐
Wealth tercile							
Lowest wealth	82.6 (95)	1.60	(0.93, 2.77)	0.241	‐	‐	‐
Middle wealth	85.4 (152)	1.31	(0.70, 2.45)		‐	‐	‐
Highest wealth	77.7 (157)	1			‐	‐	‐

*Note*. 95% CI, 95% confidence interval; BMS, breastmilk substitute; CPCF, commercially produced complementary food; OR, odds ratio.

a All analyses utilize logistic regression controlled for clustering at the health facility.

b Unadjusted odds ratios, 95% confidence intervals, and *p*‐values from bivariate analysis.

c All factors significant at *p* ≤ 0.05 in bivariate analysis were included in the full adjusted multivariable model. Adjusted odds ratio, 95% confidence intervals, and *p*‐values from the full model are reported. The dashes indicate that the variable was not included in the full model.

d *p*‐value reported for categorical variables is the *p*‐value for the overall variable, not individual categories.

**Table 4:** Bivariate and multivariable logistic regression of sugar‐sweetened beverage consumption in the previous day among children 6–35 months^a^ (*n* = 495)

Variable	SSB consumed	Unadjusted bivariate^b^			Adjusted multivariable^c^		
	% (*n*)	OR	(95% CI)	*p*‐value^d^	OR	(95% CI)	*p*‐value^d^
**Maternal factors**							
Maternal age (year)		1.01	(0.98, 1.04)	0.599	‐	‐	‐
Parity							
Primaparous	36.4 (68)	1			‐	‐	‐
Multiparous	42.2 (130)	1.28	(0.91, 1.80)	0.160	‐	‐	‐
Maternal education							
Elementary, Junior high	42.7 (44)	1.41	(0.83, 2.38)	0.113	‐	‐	‐
Senior High	43.2 (92)	1.43	(1.01, 2.03)		‐	‐	‐
Diploma, University	34.6 (62)	1			‐	‐	‐
Any work in past month							
Yes	35.3 (54)	0.75	(0.48, 1.16)	0.198	‐	‐	‐
No	42.1 (144)	1			‐	‐	‐
Hours away from child preceding day							
0	42.0 (173)	1			‐	‐	‐
>0 to <8	33.3 (10)	0.69	(0.29, 1.66)	0.064	‐	‐	‐
≥8	28.3 (15)	0.55	(0.33, 0.91)		‐	‐	‐
Reported exposure to SSB promotion							
Yes	40.9 (194)	2.94	(0.55, 15.64)	0.205	‐	‐	‐
No	19.1 (4)	1			‐	‐	‐
**Child factors**							
Child age (months)							
6.0–11.9	2.0 (2)	0.07	(0.02, 0.31)	<0.001	0.09	(0.02, 0.38)	<0.001
12.0–17.9	22.2 (22)	1			1		
18.0–23.9	49.5 (49)	3.43	(2.22, 5.31)		3.56	(2.29, 5.55)	
24.0–29.9	65.7 (65)	6.69	(3.46, 12.93)		5.47	(2.36, 12.69)	
30.0–35.9	60.6 (60)	5.38	(3.13, 9.26)		3.94	(1.83, 8.37)	
Child sex							
Male	40.3 (108)	1.03	(0.77, 1.37)	0.854	‐	‐	‐
Female	39.7 (90)	1			‐	‐	‐
Sick preceding day							
Yes	38.7 (121)	0.86	(0.57, 1.29)	0.467	‐	‐	‐
No	42.3 (77)	1			‐	‐	‐
Amount ate preceding day versus normal							
Same amount	42.0 (76)	1			‐	‐	‐
Less food	39.0 (94)	0.88	(0.64, 1.21)	0.740	‐	‐	‐
More food	38.4 (28)	0.86	(0.49, 1.51)		‐	‐	‐
Ate different foods preceding day versus normal							
Yes	40.8 (49)	1.05	(0.65, 1.68)	0.849	‐	‐	‐
No	39.7 (149)	1			‐	‐	‐
Breastfed preceding day							
Yes	29.0 (79)	1			1		
No	53.4 (119)	2.80	(1.79, 4.35)	<0.001	1.88	(0.88, 4.05)	0.105
BMS preceding day							
Yes	33.5 (82)	0.58	(0.42, 0.81)	0.001	0.31	(0.20, 0.47)	<0.001
No	46.4 (116)	1			1		
CPCF preceding day							
Yes	21.1 (39)	0.25	(0.19, 0.33)	<0.001	0.53	(0.37, 0.76)	0.001
No	51.3 (159)	1			1		
**Household factors**							
Number household members		0.96	(0.88, 1.06)	0.431	‐	‐	‐
Wealth tercile							
Lowest wealth	40.6 (65)	1.21	(0.77, 1.90)	0.208	‐	‐	‐
Middle wealth	43.7 (69)	1.37	(0.97, 1.94)		‐	‐	‐
Highest wealth	36.2 (64)	1			‐	‐	‐

*Note*. 95% CI, 95% confidence interval; BMS, breastmilk substitute; CPCF, commercially produced complementary food; OR, odds ratio; SSB, sugar‐sweetened beverage.

a All analyses utilize logistic regression controlled for clustering at the health facility.

b Unadjusted odds ratios, 95% confidence intervals, and *p*‐values from bivariate analysis.

c All factors significant at *p* ≤ 0.05 in bivariate analysis were included in the full adjusted multivariable model. Adjusted odds ratio, 95% confidence intervals, and *p*‐values from the full model are reported. The dashes indicate that the variable was not included in the full model.

d *p*‐value reported for categorical variables is the *p*‐value for the overall variable, not individual categories.

## 4 DISCUSSION

This study found high prevalence of consumption of commercially produced snack foods among children 6–35 months in Bandung City, with consumption often occurring frequently throughout the day and week. Consumption of these snacks begins at an early age, with almost half of children 6–11 months eating a snack food in the preceding day and rates increasing in the second year of life to about 90% and then remaining high. Previous research has documented snack consumption among Indonesian children, though these studies employed a broad definition of *snack* including both commercial and traditional/homemade snacks. Our study is a first to focus specifically on consumption of commercial snack foods, including types and frequency, among children under three years of age. Although we did not collect data on quantity of snacks consumed, our results reflect a high presence of commercially produced snack products in the diets of these children during the critical complementary feeding period. Research among urban children 6–23 months in Nepal and Cambodia found similarly high consumption of commercial snack foods (74.1% and 55.0%, respectively), including among children 6–11 months (57.7% and 38.4%; Pries, Huffman, Mengkheang, et al., 2016; Pries, Huffman, Adhikary et al., 2016; Pries et al., 2017).

Sugary snacks, including biscuits and candy, were the most common types of commercial snack foods consumed in this study. A similar preference for sweetened biscuits was seen in Kathmandu Valley, where 56.6% and 84.6% of 6‐ to 23‐month‐olds consumed them in the day and week preceding the survey, respectively (Pries, Huffman, Adhikary et al., 2016), and Huffman, Piwoz, Vosti, and Dewey (2014) found 34–68% of children 6–23 months consumed sugary snack foods on the previous day in five Asian countries. Consumption of sweetened milks, many containing 12–19 grams of added sugar per single‐serving package (200–250 ml; Green, unpublished observation) was substantial in the study. Ready‐to‐drink packages of milk are popular snacks for young Indonesian children (Muslimatun & Wiradnyani, 2016) and flavoured/sweetened milks are perceived to be healthier than other SSB and bring the same benefits as milk consumption (Thomson et al., 2017). Although our study collected the reason why mothers fed SSB, we did not ask about motivations for the individual categories of SSB, so we are unable to make conclusions on mothers' perceived healthiness of sweetened milks.

The popularity of these sugary snack foods and beverages among the infants and young children in our sample is concerning. Over‐consumption of added sugars early in life can set taste preferences (Beauchamp & Menella, 2009; Ventura & Mennella, 2011) and increase the risk of overweight/obesity in childhood and development of diabetes, cardiovascular disease, and other chronic conditions later in life (de Ruyter, Olthof, Seidell, & Katan, 2012; Ebbeling et al., 2012; Malik et al., 2010; Malik, Pan, Willet, & Hu, 2013; Morenga, Mallard, & Mann, 2013; Singh et al., 2015; Vartanian et al., 2007). A systematic review of added sugar intake also found high consumption linked to lower micronutrient intake, particularly when the added sugar comes from sugar‐sweetened foods and beverages (Gibson, 2007).

Multivariable analysis highlighted factors significant in the consumption of commercial snack foods and SSB among children in this study. Maternal education level was inversely associated with consumption of a snack food in the prior day, which has been found in Nepal, Senegal, and Brazil (Gatica, Barros, Madruga, Matijasevich, & Santos, 2012; Pries et al., 2017). Although other studies found both positive and negative associations of household wealth or socioeconomic status with snack consumption (Anderson et al., 2008; Gatica et al., 2012; Huffman et al., 2014; Monteiro et al., 2011; Pries, Huffman, Adhikary et al., 2016), this study found no significant effect, possibly a result of the majority of the sample being of higher wealth status, based on the 2012 IDHS national wealth levels. Commercial snack food and SSB consumption were significantly associated with increasing child age and a recent systematic review of the contribution of snack products to diets and nutritional status of children under two years found total energy intake from snack foods and SSB increased with age throughout the complementary period (Pries, Filteau, & Ferguson, 2019). The inverse associations of BMS and CPCF consumption with consumption of snack foods and/or SSB may reflect different choices caregivers are making on which commercial products to feed as snacks.

Child preference and demand was a prominent factor in why children received commercial snacks in our study and this driving influence is in line with previous research exploring caregivers' decision‐making around child feeding in the Asia region (Pries et al., 2017; Rahman et al., 2016). Researchers investigating feeding motivations and child‐led snacking behaviours in children under 24 months in East Java found children were given snacks whenever they desired, with few restraints, as parents felt these foods brought happiness to the child (GAIN & MOH, 2013). Consumption was also allowed close to meal times and few mothers viewed their children's freedom to snack as problematic or unhealthy. Studies have shown that child‐driven feeding practices can lead to overweight (Anzman‐Frasca, Stifter, & Birch, 2012), and consumption of snacks, particularly close to meal times, can displace consumption of nutrient‐rich foods and breastmilk (Anderson et al., 2008; Kimmons et al., 2005).

Commercial promotions of manufactured snack foods and SSB have also been shown to influence consumption behaviours, including among children (Boyland et al., 2016; Kelly et al., 2016). Snack foods and SSB are heavily advertised and promoted throughout the Asia region (Kelly et al., 2016). Our study did not find an association between snack and SSB consumption and exposure to promotions, but our sample was not powered to test an association given the ubiquitous exposure to promotions reported by mothers. There is burgeoning global interest and action by a number of countries to establish legislation limiting promotions of commercial snack foods and SSB and to enact front of package labelling, to encourage healthier choices and consumption (Chambers, Freeman, Anderson, & MacGillivray, 2015; Ducrot et al., 2016; Hersey, Wohlgenant, Arsenault, Kosa, & Muth, 2013; Kraak et al., 2016).

In Indonesia, snack consumption has been associated with significantly lower height‐for‐age *z*‐scores in schoolchildren and higher consumption was found among stunted children 6–59 months compared with nonstunted children in rural Central Java (Purwestri et al., 2018; Sekiyama et al., 2012). With the stagnant levels of child stunting, growing rates of overweight/obesity among the young, and persistent suboptimal infant and young child feeding practices, national and subnational multi‐sectoral attention must focus on developing and implementing interventions to reduce reliance on processed, high‐sugar/salt commercial snacks, and increasing consumption of healthy, nutrient‐rich, locally available foods during the complementary feeding period. Contextually relevant complementary feeding guidelines, and caregiver education and support, accompanied by policy action and restrictions on marketing, have the potential to not only improve the growth and development of Indonesian young children, but also positively offset the future burden of overweight/obesity and chronic non‐communicable diseases.

A number of limitations of this study should be noted. Health facilities were sampled as proxies to reach the general population, and our sampling frame only included facilities with 100 or more child‐visits per month. Mothers who do not seek services, or seek services at smaller/less busy facilities or at community‐based services, may differ from those in our survey. Mothers who refused to participate mainly said they did not have time, and these busy mothers may also differ. As mothers were recruited while seeking child health services, two‐thirds of children in our survey were reported sick in the previous day, which may impact appetite and diet; however, our bivariate analyses showed child illness, amount consumed in the preceding day, and consumption of different foods in the preceding day had no significant impact on likelihood of snack consumption. Another limitation is that our study interviewed only mothers (thus excluding children with other caregivers) who may have different feeding practices or other characteristics. A number of mothers were away from their children for work or other reasons, which could result in gaps in their diet recall. We made efforts to contact other caregivers to complete diet information, but for 2.9% complete diet data could not be collected. We added prompted questions about consumption of all commercial products to improve recall, but dietary data are subject to recall bias. Daily frequency of consumption was based on the number of times a child ate a snack product during the previous day, but no measures of quantity were taken so the study is unable to make conclusions on the contributions these products may have had on the children's diets.

## CONFLICTS OF INTEREST

The authors declare that they have no conflicts of interest.

## CONTRIBUTIONS

SH, EZ, and AP conceptualized and designed the study with input from DH and DI. MG oversaw questionnaire development, data management, analyzed the data, and prepared the manuscript. DH, MG, and AP oversaw enumerator training, and DH oversaw data collection. All authors reviewed and provided input on the final article.
